# A special needs dentistry study of institutionalized individuals with intellectual disability in West Sumatra Indonesia

**DOI:** 10.1038/s41598-019-56865-2

**Published:** 2020-01-13

**Authors:** Valendriyani Ningrum, Wen-Chen Wang, Hung-En Liao, Abu Bakar, Yin-Hwa Shih

**Affiliations:** 1 Department of Healthcare Administration, Asia University, 500, Lioufeng Rd., Wufeng, Taichung 41354 Taiwan; 2 School of Dentistry, Baiturrahmah University, By pass km 15 Aie Pacah, Padang, West Sumatra Indonesia; 3 Division of Oral Pathology & Maxillofacial Radiology, Kaohsiung Medical University Hospital, No.100, Tzyou 1st Road, Kaohsiung, 807 Taiwan

**Keywords:** Dental public health, Psychology and behaviour, Risk factors

## Abstract

People with intellectual disability have a higher risk of oral health problems. This study assessed the clinical oral health status and behaviors and treatment needs of people living in an institution in Padang, West Sumatra, Indonesia. We quantified oral health status of 65 individuals with intellectual disability using Oral Hygiene Index Simplified (OHIs), Angle’s classification of malocclusion, Community Periodontal Index and Treatment Need (CPITN), and decay index and also recorded their brushing behavior. We found that males had significantly lower OHIs (p < 0.001), more malocclusion (p < 0.001), greater caries number (p < 0.001), greater CPITN (p = 0.001) and higher need of dental treatment (p < 0.01) than females. Additionally, we found that high caries number was associated with poor OHIS, malocclusion, periodontal disease, and dependent brushing behavior (p < 0.001). The findings of this study imply that there is a gap in appropriate oral health care in individuals with intellectual disability. There should be a greater focus on providing appropriate oral health education to people with intellectual disability, improving the health literacy and quality of care of caregivers, and providing more dentists with specialized training in special needs dentistry.

## Introduction

Intellectual disability (ID) is a neurodevelopmental disorder. People with ID have impaired intellectual and adaptive functioning with an intelligence quotient (IQ) below 70. The American Association on Intellectual and Developmental Disabilities (AAIDD) divides ID into four categories according to IQ: mild (55–69), moderate (36–54), severe (20–35), and profound (<20). People with ID have difficulty in understanding, learning, and applying new or complex skills. They experience a higher risk of health problems due to an inability to properly perform activities of daily living (ADL)^1^. However, the secondary health problems created by low ADL capacity can be prevented by improving the environment and skills of these individuals, developing caregivers’ health literacy, and strengthening the special needs training of medical professionals involved with their care.

Systematic reviews and meta-analyses on the oral health status of people with ID reveal the poor oral health of these individuals and the great need for dentistry practices geared to them^2,3^. Individuals with ID encounter oral health problems, such as caries^4^, poor oral hygiene^5^, periodontal disease^6,7^, and malocclusion^4^. Children with ID have high DMF-T (decay, missing, filling-tooth) scores^4^. It has been reported that 44.3% of people with ID had signs of gingival disease^7^, while 84.8% have incisal segment crowding^4^. The prevalence of malocclusion in children with Down’s syndrome and cerebral palsy is 20.4%; among these individuals, 21.5% have anterior crossbite, 21.5% have posterior crossbite, and 29.8% have anterior open bite^8^. Several risk factors cause secondary oral health problems in people with ID. These include low ADL capacity^9^, which makes them unable to brush the teeth adequately^6^, favoring cariogenic foods^10^, and lack of special health education training.^11^ The inadequate quality of care given by caregivers^12^ and lack of special needs dentistry training of dental professionals also contribute to this issue^13^. In addition, the oral health perception of specialized institutions affects the oral health status of people who live there^14^.

Few papers discuss the current oral health care needs of institutionalized individuals with ID in Indonesia. As a basis for improving oral health care and quality of life in this population, this study assessed the clinical oral health status of individuals with ID living in the only institution in Padang, West Sumatra Indonesia geared towards them.

## Results

### Participant characteristics

The demographics of the participants are listed in Table 1. This study enrolled 65 participants with ID, 34 females and 31 males. The mean age of the group was 22 years. Most participants had a moderate degree of intellectual disability (87.7%) while the remaining 12.3% of individuals had a mild degree of intellectual disability. None of the participants had a severe ID.

**Table 1:** The demographic data of the study participants.

Character	Gender			
	Female N = 34		Male N = 31	
	N	%	N	%
**Degree of Intellectual disability**				
Mild	4	11.8	4	12.9
Moderate	30	88.2	27	87.1
**Age group**				
Adolescent (13y–18y)	9	26.5	11	35.5
Young adult (19y–24y)	15	44.1	11	35.5
Adult (25y–45y)	10	29.4	9	29.0
**Mean age (±SD)**	22 (±4.916)		22 (±5.891)	

### Differences in oral health status between the sexes

Oral health status, brushing behavior, and dental treatment needs were determined separately for males and females **(**Table 2**)**. The parameters of oral health status included OHIs, occlusion classification, and CPITN. A higher proportion of males than females had poor OHI (90.3% vs. 0%), class 2 or 3 malocclusion (96.7% vs. 23.5%), and periodontal bleeding or calculus (100% vs. 73.5%). Brushing ability was measured in terms of whether individuals could brush their teeth independently and the number of times they brushed their teeth per day. All females (100%), but not all males (41.9%), brushed their teeth independently, either once or twice a day. Treatment needs in terms of scaling, restoration, and orthodontic treatment, were determined. The male participants showed significantly greater treatment needs than females in all three items: scaling (100% vs. 73.5%) (p < 0.001), restoration (87.1% vs. 58.8%) (p < 0.05), and orthodontic treatment (96.8% vs. 23.5%) (p < 0.001). Males had a significantly higher mean caries number than females (7.55 mean caries number vs. 3.5 mean caries number, p < 0.001) **(**Table 3**)**. Thus, the oral health status, occlusion classification, and brushing ability was significantly worse in males than in females, and male participants required more dental treatment.

**Table 2:** Comparison of the oral health status, brushing behavior, and dental treatment needs between male and female study participants.

Items	Variable		Gender				*p*
			Female N = 34		Male N = 31		
			N	%	N	%	
Oral health status	OHIS	Good	2	5.9	0	0.0	<0.001***
		Fair	32	94.1	3	9.7	
		Poor	0	0.0	28	90.3	
	Occlusion	Class 1	26	76.5	1	3.2	<0.001***
		Class 2	8	23.5	21	67.7	
		Class 3	0	0.0	9	29.0	
	CPITN	Healthy	9	26.5	0	0.0	0.001**
		Bleeding on Probing	24	70.6	23	74.2	
		Calculus	1	2.9	8	25.8	
		Pocket 4–5 mm	0	0.0	0	0.0	
		Pocket >6 mm	0	0.0	0	0.0	
Brushing behavior	Brushing capability	Independent	34	100.0	13	41.9	<0.001***
		Dependent	0	0.0	18	58.1	
	Brushing Frequency	Once a day	18	52.9	0	0.0	<0.001***
		Twice a day	16	47.1	31	100.0	
Dental treatment needs	Scalling Needs	No	9	26.5	0	0.0	0.002**
		Yes	25	73.5	31	100.0	
	Restoration Needs	No	14	41.2	4	12.9	0.011*
		Yes	20	58.8	27	87.1	
	Orthodontic Needs	No	26	76.5	1	3.2	<0.001***
		Yes	8	23.5	30	96.8	

Chi-squared test *p < 0.05, p** < 0.005, ***p < 0.001.

**Table 3:** The mean caries number of the individuals with ID.

	Caries number			
	N	Mean	SD	*p*
Female	34	3.00	1.923	<0.001***
Male	31	7.55	2.862	

unpair t-test ***p < 0.001.

### Independent predictors of caries and their correlation

Stepwise multiple linear regression analysis revealed that age, oral hygiene, periodontal disease, malocclusion (class 2 and class 3), brushing capacity, and brushing frequency were predictors of the caries number (p < 0.001). The increased adjusted R square indicated the independent variables predict the caries number (Table 4). Table 5 shows the coefficients of the multiple linear regression analysis. The variance inflation factors were less than 10 indicating the absence of multicollinearity between independent variables.

**Table 4:** Multiple linear regression analysis of association factors to dental caries.

Model	R	R Square	Adjusted R Square	Std. Error of the Estimate	*p* value
a	0.840	0.706	0.701	1.813	<0.001***
b	0.888	0.789	0.782	1.549	<0.001***
c	0.928	0.861	0.854	1.267	<0.001***
d	0.938	0.880	0.872	1.185	<0.001***
e	0.947	0.897	0.888	1.109	<0.001***
f	0.951	0.904	0.895	1.076	<0.001***
g	0.950	0.902	0.894	1.079	<0.001***

a. Predictors: (Constant), Occlusion.

b. Predictors: (Constant), Occlusion, OHIS.

c. Predictors: (Constant), Occlusion, OHIS, Age.

d. Predictors: (Constant), Occlusion, OHIS, Age, Brushing capacity.

e. Predictors: (Constant), Occlusion, OHIS, Age, Brushing capacity, CPITN.

f. Predictors: (Constant), Occlusion, OHIS, Age, Brushing capacity, CPITN, Brushing Frequency.

g. Predictors: (Constant), OHIS, Age, Brushing skill, CPITN, Brushing Frequency.

**Table 5:** The coefficient of the multiple linear regression analysis.

Model	Unstandardized Coefficients		Standardized Coefficients	t	Sig.	Collinearity Statistics	
	B	Std. Error	Beta			Tolerance	VIF
(Constant)	2.277	0.325		6.996	<0.001		
Occlusion	4.000	0.325	0.840	12.288	<0.001	1.000	1.000
(Constant)	−0.218	0.577		−0.377	0.708		
Occlusion	2.499	0.412	0.525	6.063	<0.001	0.455	2.197
OHIS	2.557	0.519	0.427	4.931	<0.001	0.455	2.197
(Constant)	−4.856	0.950		−5.112	<0.001		
Occlusion	1.432	0.387	0.301	3.702	<0.001	0.346	2.892
OHIS	3.353	0.447	0.560	7.499	<0.001	0.410	2.441
Age	0.191	0.034	0.310	5.627	<0.001	0.751	1.331
(Constant)	−3.839	0.947		-4.055	<0.001		
Occlusion	1.114	0.376	0.234	2.964	0.004	0.320	3.122
OHIS	2.816	0.452	0.470	6.230	<0.001	0.350	2.854
Age	0.171	0.032	0.277	5.251	<0.001	0.720	1.389
Brushing skill	1.548	0.496	0.211	3.120	0.003	0.438	2.281
(Constant)	−3.439	0.896		−3.840	<0.001		
Occlusion	0.647	0.383	0.136	1.688	0.097	0.270	3.702
OHIS	2.592	0.429	0.433	6.036	<0.001	0.340	2.939
Age	0.123	0.034	0.199	3.601	0.001	0.572	1.750
Brushing skill	1.625	0.465	0.221	3.495	0.001	0.437	2.288
CPITN	1.301	0.423	0.208	3.078	0.003	0.382	2.617
(Constant)	−3.142	0.880		−3.570	0.001		
Occlusion	0.431	0.385	0.091	1.119	0.268	0.252	3.972
OHIS	2.291	0.440	0.382	5.212	<0.001	0.306	3.269
Age	0.108	0.034	0.175	3.182	0.002	0.547	1.829
Brushing skill	1.887	0.467	0.257	4.038	<0.001	0.408	2.454
CPITN	1.180	0.414	0.189	2.848	0.006	0.375	2.667
Brushing Frequency	0.926	0.430	0.126	2.154	0.035	0.481	2.078
(Constant)	−3.433	0.842		−4.076	<0.001		
OHIS	2.456	0.415	0.410	5.916	<0.001	0.344	2.903
Age	0.111	0.034	0.180	3.289	0.002	0.551	1.814
Brushing skill	2.054	0.444	0.279	4.628	<0.001	0.454	2.203
CPITN	1.339	0.390	0.214	3.433	0.001	0.425	2.352
Brushing Frequency	1.051	0.416	0.143	2.527	0.014	0.516	1.937

Dependent Variable: Caries.

VIF: variance inflation factor.

### Participants who had poor oral health status and dependent brushing capacity are at high risk to have caries

Simple odds ratios were calculated to determine the association of different components of brushing behavior and oral health on dental caries **(**Table 6**)**. Poor OHIS status increased the odds of developing caries 66.1 times while an inability to brush teeth independently increased the odds of developing caries 5.8 times.

**Table 6:** The odds ratio of the caries among individuals with ID.

Variables	Odds ratio	95% CI
Poor OHIS	66.11	2.74-1596.19
Malocclusion	23.27	1.25–433.57
Periodontal disease	209.85	9.72–4531.71
Dependent brushing behavior	5.79	0.31–108.34
Once a day brushing behavior	49.4	2.60–937.44

## Discussion

The quality of life of individuals with ID can markedly improve by addressing issues regarding their oral health. However, few papers on the oral health of individuals with ID in Indonesia exist. Therefore, we assessed the clinical oral health status of people with ID living in specialized institutions in Indonesia. We found that males had significantly worse health status and required more extensive dental treatment than females. Additionally, we found that different aspects of brushing teeth and oral health such as ability to brush one’s teeth independently and periodontal health significantly affected caries number.

The participants in the present study had mild to moderate ID. This is because the institution in Padang does not admit individuals with severe ID^15^. In our study, poor oral health status and malocclusion were more prevalent in males than in females, and males more often required assistance to brush their teeth. This may be because, in individuals with ID, males exhibit more responsive behavior and have a higher prevalence of visual impairments, like strabismus and refractive errors, than females^16–18^.

Individuals with ID have deficits in mental abilities, social skills, and lower ADL ability compared to their same-aged peers. Approximately one-fourth of cases of ID may be attributable to genetic causes and the indelible nature of the condition makes it necessary for health care workers and caregivers to be more accommodating to them, particularly when promoting self-care. Individuals with ID may take longer to learn to brush their teeth and rinse their mouth. Some of them are unable to chew and swallow food adequately, and thus food debris accumulates in the oral cavity. Furthermore, the prevalence of enamel defects and malocclusion is high in individuals with ID^19,20^.

The main oral health problems in the ID group are periodontal disease, caries, and malocclusion. Due to inadequate chewing and swallowing, and consequent food debris accumulation in the oral cavity, as well as poor self-brushing capacity, periodontal disease^3,21,22^ and caries frequently occur in individuals with ID^17^. Periodontal disease could be caused by the accumulation of dental plaque as well as a hyperactive immunological response. Environmental stress could further exacerbate this immunological response. Residing in this institution may be more stressful for these individuals than living at home and attending a special school because family time may be restricted. Additionally, because there is only 1 caregiver per 7 individuals, there may be insufficient care, particularly oral care, increasing the chances of periodontal disease and caries. It has been reported that individuals living in such institutions are more prone to having periodontal disease and that this predilection is reduced after being de-institutionalized^23^. The study population had a high prevalence of malocclusion as well as dental caries. The acquired enamel defects can have an impact on caries development^19^ and tooth loss^24,25^. Previous literature has shown that caries development in adults with physical and intellectual disabilities is associated with diet, frequency of dental visits, absence of oral hygiene assistance, career-contact hours, and transportation problems^26^. We found that in this population, malocclusion, OHIS, age, CPITN, brushing fewer than two times per day, and inability to brush one’s teeth independently correlated with caries number. Increasing or altering oral hygiene assistance in individuals that cannot brush their teeth independently may decrease caries numbers.

We consider that improving the oral health status among individuals with ID can be grouped into three categories: improving health education methods and creating a friendly environment for individuals with ID, enhancing oral health literacy and quality of care among caregivers, and establishing a special needs dentistry course for dentists who would like to treat individuals with ID.

In this special care institution in Padang, the caregivers teach residents how to bathe. Residents have to rely on oral hygiene skills acquired from their family members as they were growing up. This is because this institution does not formally train caregivers to promote oral hygiene. To enhance oral health skills among individuals with ID, dental health education can be taught through a peer- mediated time-delay approach where peers model appropriate brushing techniques and habits and prompt individuals with ID to carry out this behavior. This has been shown to be an effective approach to teaching science in such individuals^27^. In order to promote better oral hygiene habits, a brief motivational interview (lasting 15–20 minutes) can be conducted prior to dental education. Rating performance, and providing feedback on daily toothbrushing and oral health are feasible means to improve education^28^. Additionally, audiotapes can be used to consolidate the information that is taught.

Financial barriers and a lack of parental awareness are major factors preventing individuals with ID to receive dental care^21,25,29^. The poor oral health status of people with ID has been associated with poverty^6,22^. Overcoming this financial barrier by social insurance should be considered. The responsive behavior of people with ID presents a further challenge to normal dental treatment procedures^30^. The teaching-family model can help to reduce physical and verbal aggression^31^.

According to previous reports, 65% of people with ID are under the supervision of a caregiver^32^. The lack of understanding of the importance of oral health and inadequate ADL support from the caregiver can increase the predilection of oral health problems in people with ID^33^. Appropriate support for brushing teeth more than once a day among people with ID could significantly reduce periodontal disease and caries indices^34–38^. This can only occur with increasing the oral health literacy of caregivers^39^.

The findings of this study are not generalizable to all individuals with ID and may only apply to institutionalized individuals with ID in Indonesia. Additionally, this study did not look at other factors that are more common in individuals with ID, which may affect oral health like bruxism, tongue thrusting, and responsive behavior. None the less, this study may offer some insight on the necessity to enhance special needs dentistry in Indonesia. Dental care significantly improves the oral health and quality of life of individuals with ID^40^. However, special needs dentistry is a challenging dental field. Dentists need to be equipped with adequate knowledge and skills to address the special needs of these patients. Treatment and admission protocols that do not accommodate these individuals and an unfriendly environment lower the quality of care for these individuals^41^. Therefore, a special training course for people involved in dental care should be established where special needs dentistry units are lacking, to train dentists who will devote themselves to dental care of individuals with special needs.

## Materials and Methods

### Ethical considerations

This study was approved by the committee of the research ethics of the faculty of medicine, Andalas University in Padang with the approved number of 014/KEP/FK/2019. Written informed consent was obtained from the guardians of participants with ID, and those ID participants who were willing to join this study. Consent to publish open access journal was obtained from guardians. The information letter describing the rationale of the study and individual rights was handed to the guardians. All clinical survey was performed in accordance with the guidelines of the Declaration of Helsinki.

### Study participants

The participants were residents at the only institution for the mentally disabled in Padang, West Sumatra, Indonesia. The ID classification data of each participant were provided by the school Sixty-five participants met the inclusion criterion of good general health (ASA Physical Status Classification I and II)^42^. Exclusion criteria were as follows: parents declined participation, systemic diseases, and responsive behavior.

### Oral examination

The oral examination was performed by five well-trained examiners. The reliability of examiners was assessed based on the data of seven participants. The intraclass coefficients for intra-examiner and inter-examiner reliabilities were 1 and 0.91, respectively^10^. All oral assessments were performed in daylight in the school classrooms using flashlights for further visualization. During the assessment, the study participant was seated on a chair. Meticulous oral examination was carried out with a guardian assisting when necessary to provide comfort to the participant. During the examination, a disposable dental kit (sonde, mirror, and excavator) and BPWHO dental probe (Osung, Pearland, TX, USA) was used. Tooth-brushing behavior was determined by asking participants questions about the number of times they brushed their teeth per day (tooth brushing frequency) and whether they required assistance from their caregivers when brushing their teeth (tooth brushing skills). The guardians assisted with communication during the interview process.

### Oral hygiene index

Oral hygiene was quantified using the simplified oral hygiene index (OHI)^43^. The OHI has two components, the debris index simplified, and the calculus index simplified. Six surfaces were examined to determine this score: teeth 16, 11, 26, 36, 31, and 46. Oral hygiene was classified as good (score: 0–1.2), fair (1.3–3), and poor (3.1–6).

### Caries number

The caries number was scored using the oral health survey of the World Health Organization (WHO) (2013). The caries number was expressed as the total number of teeth that were decayed in an individual. When caries or both caries and a restoration were present, the tooth was recorded as decayed.

### Community periodontal index

The community periodontal index of treatment needs (CPITN) was examined according to the WHO methodology (1997)^44^. The CPITN procedure employed a WHO probe and mouth mirror. The dentition was divided into six sextants: Upper right (tooth 17 to 14), upper anterior (tooth 13 to 23), upper left (tooth 24 to 27), lower right (tooth 47 to 44), lower anterior (tooth 43 to 33), and lower left (tooth 34 to 37). The probe was moved around the gingival sulcus, and the highest score recorded for each sextant was scored, as follows:

0 = healthy 1 = bleeding during probing 2 = supra- and/or subgingival calculus, filling or crown excesses 3 = gingival pockets 4 to 5 mm 4 = pockets deeper than 6 mm.

### Malocclusion angle’s classification

The Malocclusion Angle’s classification was based on the relationship of the first molars^45^. Class 1 malocclusion was defined when the mesiobuccal cusp of the permanent maxillary first molar approximated the buccal groove of the permanent mandibular first molar. Class 2 malocclusion was defined when the maxillary mesiobuccal cusp fell anterior to the buccal groove of the mandibular molar and class 3 define as the maxillary mesiobuccal cusp fell distal to the buccal groove of the mandibular molar. In this article, we categorized the class 2 and class 3 as positive malocclusion and class 1 as negative malocclusion.

### Statistical analysis

Data analysis was performed using the Statistical Package for the Social Sciences software version 23.0 for Windows (IBM Corp, New York, NY, USA). Continuous variables were analyzed using the *t*-test while categorical variables were analyzed using Fisher’s exact test. In order to determine whether sex, periodontal status, malocclusion, age, brushing frequency, brushing skill and oral hygiene were predictors for caries number, stepwise multiple linear regression analysis was carried out. *P* < 0.05 was considered statistically significant. In Table 4, we analyzed the multiple linear regression with several categorical variables. The reference category of occlusion is class 2 and class 3, the reference category of OHIS is fair and poor oral hygiene, the reference category of age is 26–36 years, the reference category of brushing capacity is dependent tooth brushing, the reference category of CPITN is bleeding on probing and calculus, and the reference category of brushing frequency is once a day or less.
